# Reflex therapy in patients with chronic tension-type headaches: Effectiveness via sensory and affective Mcgill pain questionnaire descriptors

**DOI:** 10.1192/j.eurpsy.2021.1985

**Published:** 2021-08-13

**Authors:** G. Shevtsova, E. Malenkova, O. Zagorulko

**Affiliations:** 1 Department Of Nervous Diseases And Neurosurgery, I.M. Sechenov First Moscow State Medical University (Sechenov University), Moscow, Russian Federation; 2 Pain Clinic, Russian Scientific Centre of Surgery named after B.V.Petrovsky, Moscow, Russian Federation

**Keywords:** acupuncture, Chronic Pain, headache, chronic tension-type headaches

## Abstract

**Introduction:**

Tension-type headaches is the most common type of headache among adults and it rises a challenge in findig an effective and safe treatment method.

**Objectives:**

The study aims to evaluate the corporal acupuncture therapy efficacy in patients with chronic tension-type headaches undergoing a complex treatment plan.

**Methods:**

The study involved 132 patients (74% female and 26% men) aged 18-65 years, who were divided into two groups. Patients reported their pain lasted 0.4-12 years. All the patients received conventional treatment (central muscle relaxants and antidepressants). The study group additionally received classical corporal acupuncture 3 times per week, a course of 12 sessions. Treatment effectiveness was evaluated by measuring pain intensity using a subjective visual analogue scale (VAS) and McGill Pain Questionnaire (MPQ).

**Results:**

Most of the patients (79% and 88% of study and control groups respectively) demonstrated moderate cervical musculoskeletal dysfunctions. At admission pain intensity was 4.2±1.5 and 3.8±1.7 VAS points in the control and study groups respectively, MPQ sensory rank pain index (RPI) was 5.92±1.49 points, affective RPI 3.41±0.84, the total RPI – 7.12±2.56 in the control group, and 6.22±1.74; 2.98±0.62 and 7.14±1.65 points in the study group. 4 weeks after treatment measurements showed following pain intensity changes: 4.21±0.74 vs 3.1±0.95 points in the control and study groups respectively. 3- and 6-month period revealed 3.1±0.57, 2.4±0.74 points and 2.1±0.62, 1.1±0.49 points in the control and study groups respectively.

**Conclusions:**

Classical corporal acupuncture course may benefit chronic tension-type headaches patients providing an effective treatment in a safe way.

**Disclosure:**

No significant relationships.

